# Incidence of Pancreatic Adenocarcinoma in the United States from 2001 to 2015: A United States Cancer Statistics Analysis of 50 States

**DOI:** 10.7759/cureus.3796

**Published:** 2018-12-28

**Authors:** Nicolas Patel, Ciril Khorolsky, Bikramjit Benipal

**Affiliations:** 1 Internal Medicine, New York University School of Medicine, New York, USA; 2 Internal Medicine, Temple University, Philadelphia, USA

**Keywords:** cancer epidemiology, pancreatic adenocarcinoma, gastroenterology

## Abstract

Introduction

Pancreatic cancer is one of the leading causes of death in both males and females in the United States. Nearly 85% of pancreatic cancer is adenocarcinoma. Given the silent disease progression of pancreatic cancer, identifying at-risk populations will help diagnose these fatal cancers as early as possible.

Methods

The United States Cancer Statistics (USCS) registry was used to obtain data for pancreatic adenocarcinoma from 2001 to 2015. The incidence analysis was stratified based on sex, race, stage, and US regional location.

Results

The overall incidence of pancreatic adenocarcinoma from 2001 to 2015 was 5.2 per 100,000 people per year. The overall incidence rates were the greatest for each stratification in males, blacks, distant disease, and in the Northeast. The incidence in blacks continued to rise with an annual percent change (APC) of 2.28 between 2001 and 2015. Between 2001 and 2006, the incidence of distant disease increased at a rapid rate (APC 5.34). However, after 2006, the incidence continued to increase but no longer at the previously rapid rate (APC 1.91). For incidence based on US regional location, the overall incidence was greatest in the Northeast and Midwest. The incidence in the South was increasing at an expeditious rate (APC 2.70).

Conclusion

In our study, we analyzed the incidence of pancreatic adenocarcinoma using data from all 50 states in the US. Our findings showed that there was a worsening incidence in blacks, those with a distant stage at diagnosis, and those in the North and Midwest. Ultimately our findings help identify at-risk populations and can contribute to improving surveillance of this deadly disease.

## Introduction

Pancreatic cancer is the fourth leading cause of cancer-related death in both males and females [1]. One of the most fatal cancers, it has a five-year relative survival rate of 8% [1-2]. Moreover, nearly 90 percent of pancreatic cancers are diagnosed after the age of 55 [3-5]. The most commonly diagnosed pancreatic cancers are adenocarcinomas, being diagnosed in 85% of pancreatic malignancies [2]. Modifiable risk factors for pancreatic adenocarcinoma include tobacco use, obesity, and diabetes [3]. Pancreatic cancer is typically a silent disease, as early stages are usually symptom-free [6]. It typically presents at later stages with progressive nonspecific symptoms that can include abdominal pain, jaundice, light-colored stool, fatigue, and weight loss [2]. Diagnosis at an early stage is difficult, as current diagnostic tests are nonspecific. With no definitive curative options, studies examining the epidemiology of pancreatic cancer is necessary to identify at-risk populations that can potentially lead to early diagnosis and treatment [6]. Previous studies have used incidence rates from the National Cancer Institute’s (NCI) Surveillance, Epidemiology, and End Results (SEER) program. The SEER database consists of 18 cancer registries and represents approximately 28% of the United States (US) population [7]. As a result, the SEER database, although intended to represent the entire US population, can underrepresent certain racial/ethnic groups and regions within the US. The United States Cancer Statistics (USCS) combines both the Centers of Disease Control and Prevention’s (CDC) National Program of Cancer Registries (NPCR) and the SEER database to include data for all 50 states and can be a superior representation of the US population [8]. In this study, we evaluated the incidence of pancreatic adenocarcinoma in all 50 states between 2001 and 2015, stratified by different risk factors.

## Materials and methods

Incidence data for pancreatic adenocarcinoma between 2001 and 2015 was obtained from the USCS database [9]. The USCS database provides official federal statistics on cancer incidence and population data for all 50 states and the District of Columbia. Cases were selected by primary site: C25.0 head of pancreas, C25.1 body of pancreas, and C25.2 tail of pancreas. International Classification of Diseases (ICD) for Oncology, 3rd edition, codes were used to extract data for adenocarcinoma (8140/3). The incidence analysis stratified data based on sex, race, stage (localized, regional, distant), and US region (Northeast, Midwest, South, and West). Race included those that were white, black, Asian or Pacific Islanders (API), and American Indian/Alaska Native. The incidence analysis used Tiwari et al.'s 2006 modifications for confidence interval (CI) [10]. The Joinpoint Regression Program (version 4.5.0.1, DigitCompass LLC, Maryland, USA) was used to generate incidence graphs and calculate the annual percent change (APC) using the least square method [11]. Incidence data are per 100,000 and were adjusted to the year 2000 US standard population. For all analysis, p<0.05 was considered statistically significant.

## Results

A total of 254,200 patient were included in the incidence analysis between 2001 and 2015 (Table 1). A total of 131,629 (51.8%) males and 122,571 (48.2%) females were included in the analysis. A total of 253,215 had an identifiable race at the time of diagnosis. Of those, 213,673 (84.4%) were white, 31,553 (12.5%) were black, 1,161 (0.5%) were American Indian/Alaska Native, and 6,828 (2.6%) were API. There was a total of 244,720 with a stage at diagnosis. Localized disease accounted for 21,497 (8.8%) pancreatic adenocarcinomas, 88,192 (36%) were regional, and 135,031 (55.2%) were distant. There was a total of 254,200 with an identifiable region within the US at the time of diagnosis. Of those, 54,143 (21.3%) were in the Northeast, 61,459 (24.2%) were in the Midwest, 89,627 (35.3%) were in the South, and 48,971 (19.2%) were in the West.

**Table 1:** Patient Characteristics

Patient characteristics		
Gender (n=254,200)		
	Count	Percent
Male	131,629	51.8%
Female	122,571	48.2%
Race (n=253,215)		
	Count	Percent
White	213,673	84.4%
Black	31,553	12.5%
Asian or Pacific Islander	6,828	2.6%
American Indian/Alaska Native	1,161	0.5%
Stage (n=244,720)		
	Count	Percent
Localized	21,497	8.8%
Regional	88,192	36.0%
Distant	135,031	55.2%
Regions (n=254,200)		
	Count	Percent
Northeast	54,143	21.3%
Midwest	61,459	24.2%
South	89,627	35.3%
West	48,971	19.2%

**Figure 1:**
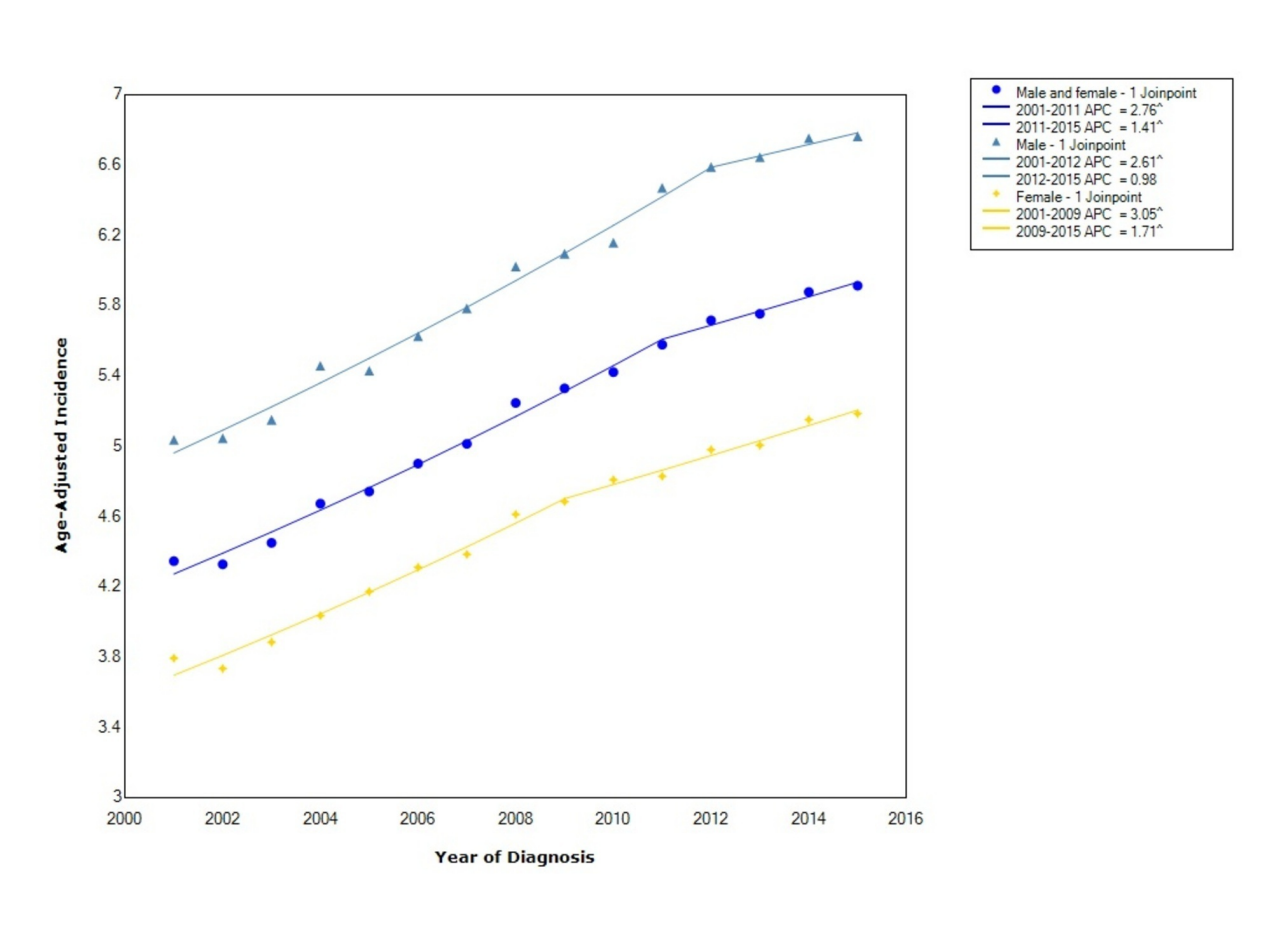
Incidence Rate, Sex APC: Annual Percent Change ^ Indicates that the APC is significantly different from zero at the alpha = 0.05 Age-adjusted incidences are per 100,000 and age adjusted to the 2000 United States standard population

The overall incidence of pancreatic adenocarcinoma from 2001-2015 was 5.2 per 100,000 people per year. Males had an incidence rate of 5.99 (95% CI 5.96-6.03), which was greater than females who had an incidence of 4.53 (95% CI 4.51-4.56). Between 2001 and 2009, the incidence in females increased with statistical significance (APC 3.05). However, after 2009, the incidence continued to increase with statistical significance but at a considerably slower rate (APC 1.71). In males, the incidence increased with statistical significance between 2001 and 2012 (APC 2.61). Similarly, after 2012, the incidence continued to rise but at a considerably slower rate (APC 0.98) (Figure 1).

**Figure 2:**
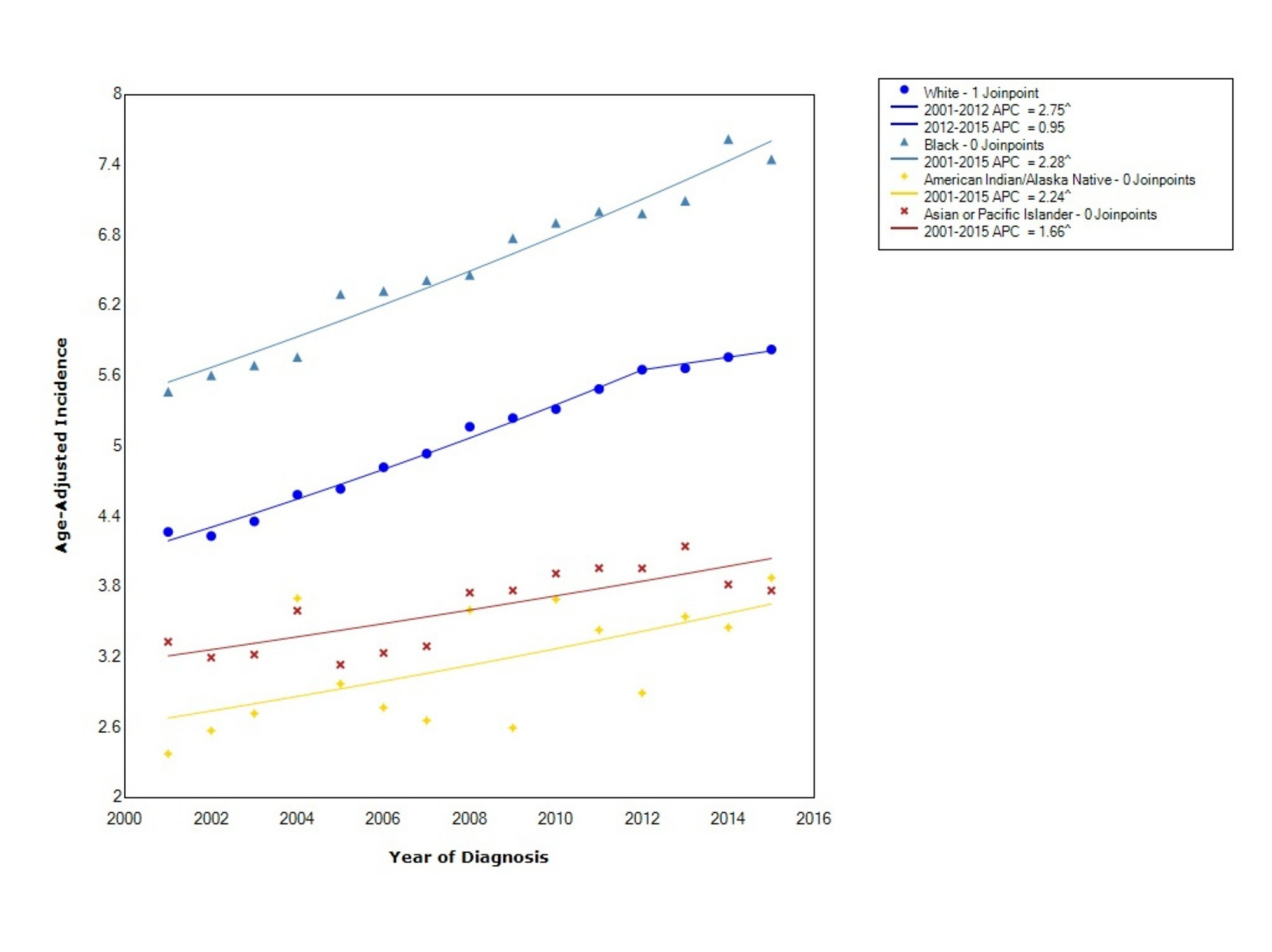
Incidence Rate, Race APC: Annual Percent Change ^ Indicates that the APC is significantly different from zero at the alpha = 0.05 Age-adjusted incidences are per 100,000 and age adjusted to the 2000 United States standard population

When stratified by race, pancreatic adenocarcinoma had the greatest overall incidence in blacks (6.6 95% CI 6.5-6.7) followed by whites (5.10 95% CI 5.08-5.12), API (3.67 95% CI 3.58-3.76), and, lastly, American Indian/Alaska Natives (3.2 95% CI 3.0-3.4). For blacks, the incidence from 2001-2015 increased at an expeditious rate, with a statistically significant APC of 2.28. The incidence in whites increased with statistical significance between 2001 and 2012 (APC 2.75); however, after 2012, the incidence increased but not at nearly the same rate (APC 0.95). The incidence in American Indian/Alaska Natives increased with statistical significance from 2001 to 2015 with an APC of 2.24. The incidence in API also rose between 2001 and 2015 with statistical significance (APC 1.66) (Figure 2).

**Figure 3:**
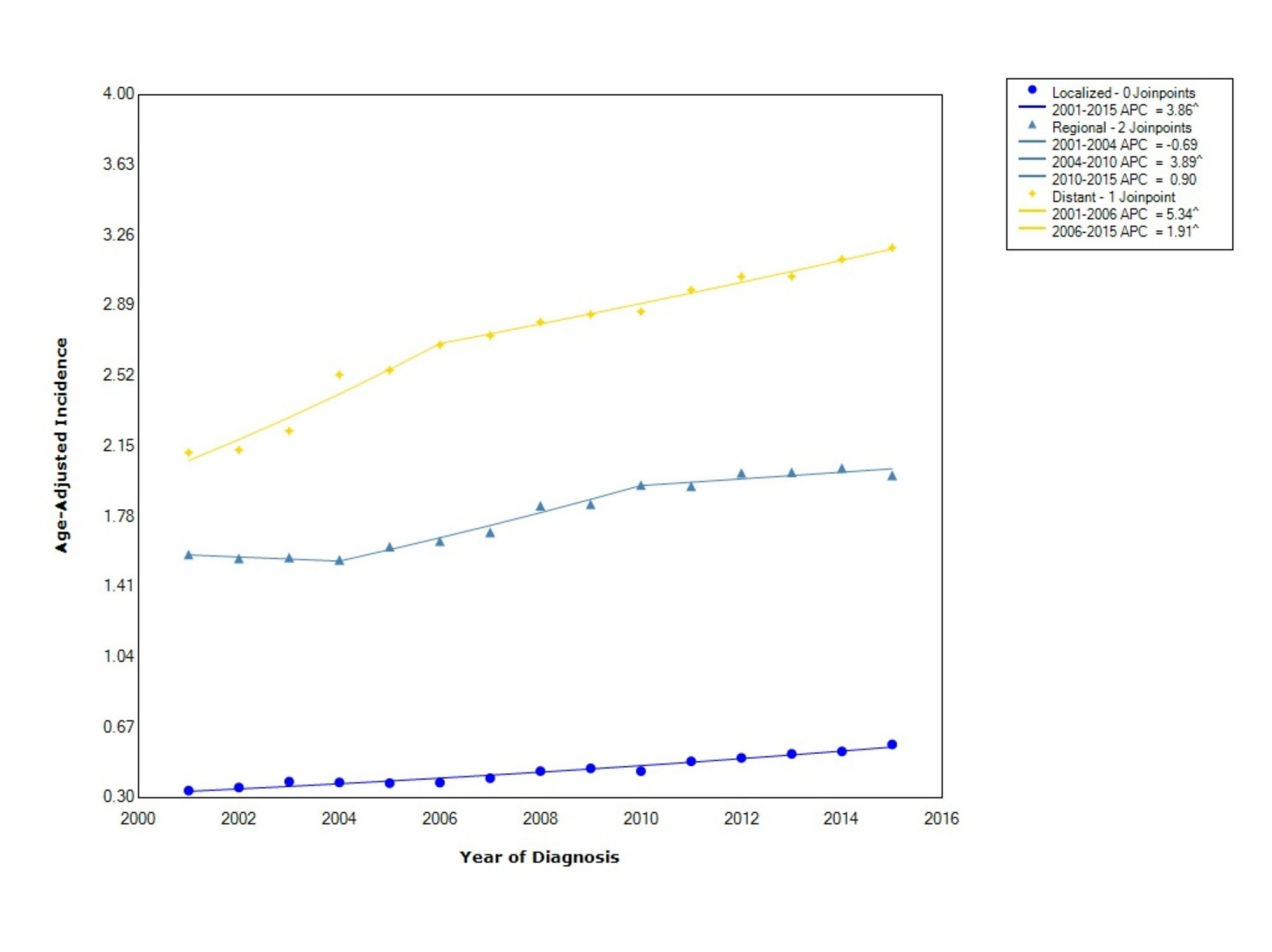
Incidence Rate, Stage APC: Annual Percent Change ^ Indicates that the APC is significantly different from zero at the alpha = 0.05 Age-adjusted incidences are per 100,000 and age adjusted to the 2000 United States standard population

Pancreatic adenocarcinoma, when stratified by stage at diagnosis showed that the incidence is greatest for distant disease (2.75 95% CI 2.74-2.77), followed by regional disease (1.80 95% CI 1.79-1.81) and localized disease (0.445 95% CI 0.440-0.452). The incidence of distant disease increased at a rapid rate (APC 5.34) between 2001 and 2006; however, after 2006, the incidence continued to rise but no longer at the same rate (APC 1.91). Regional disease, between 2001 and 2004, decreased in incidence (APC -0.69), then started to increase with statistical significance between 2004 and 2010 (APC 3.89), and then leveled off with an APC of 0.90. Localized disease increased at a statistically significant rate from 2001 to 2015 with an APC of 3.86 (Figure 3).

**Figure 4:**
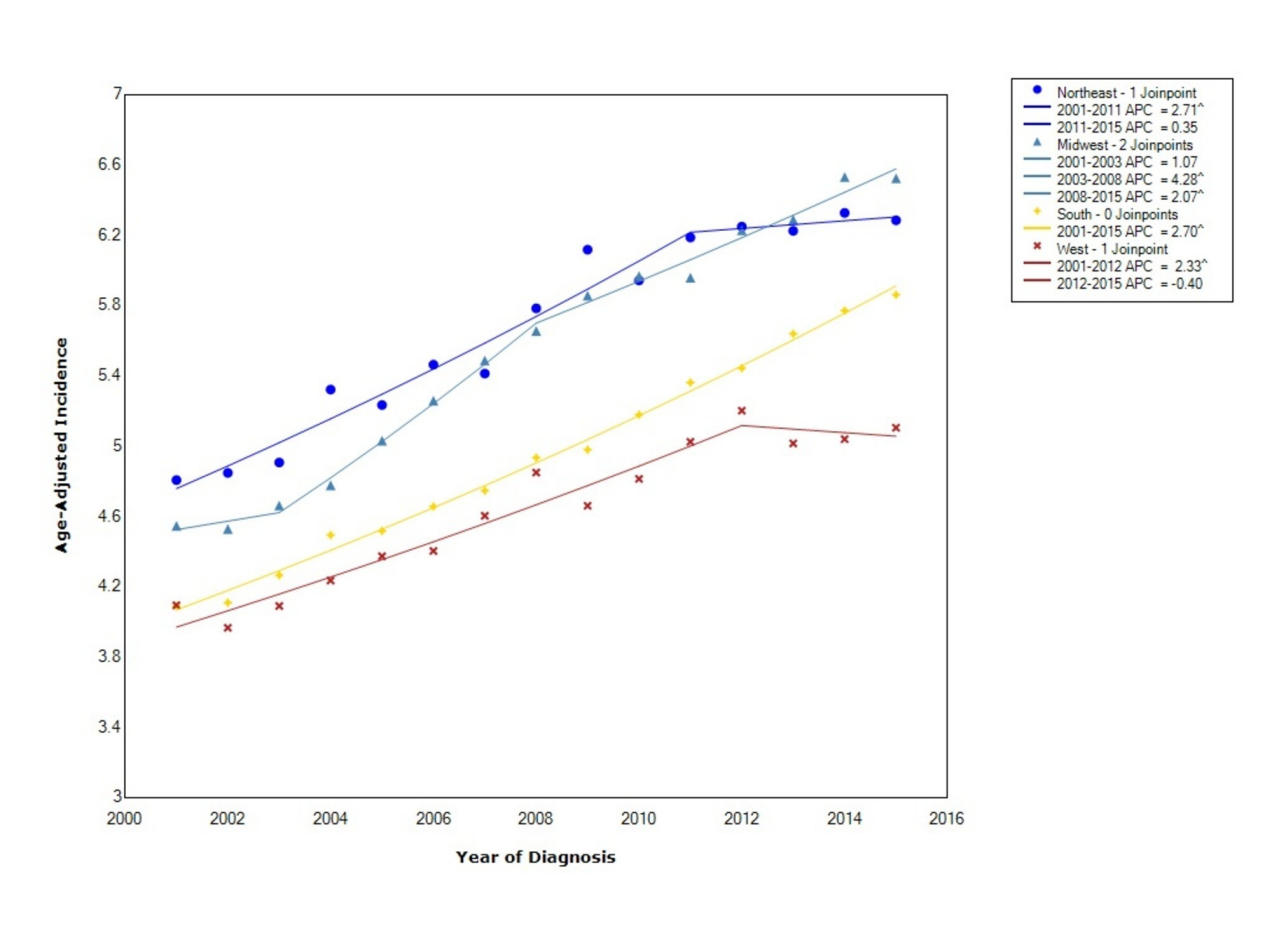
Incidence Rate, Region APC: Annual Percent Change ^ Indicates that the APC is significantly different from zero at the alpha = 0.05 Age-adjusted incidences are per 100,000 and age adjusted to the 2000 United States standard population

The overall incidence when stratified by regional location within the US showed that the incidence was greatest in the Northeast (5.69 95% CI 5.65-5.74), followed by the Midwest (5.59 95% CI 5.55-5.64), the South (4.99 95% CI 4.96-5.03), and, lastly, the West (4.67 95% CI 4.63-4.71). The incidence in the Northeast initially increased at a statistically significant rate between 2001 and 2011 (APC 2.71) and then leveled off (APC 0.35). In the Midwest, the incidence increased between 2001 and 2003 (APC 1.07) but then started to increase at a more rapid, statistically significant rate between 2003 and 2008 (APC 4.28). Following 2008, the incidence continued to increase at a statistically significant rate but not as rapidly (APC 2.07). In the South, the incidence increased with statistical significance between 2001 and 2015 (APC 2.70). Lastly, in the West, the incidence initially increased between 2001 and 2012 with statistical significance (APC 2.33) and then decreased thereafter (APC -0.40). Moreover, starting in 2013, the incidence rate in the Midwest overtook the rate in the Northeast (Figure 4).

## Discussion

Our analysis showed a difference in the incidence of pancreatic adenocarcinoma for sex, race, stage, and regional location within the US. In our study, there was an overall 1.32:1 male to female incidence ratio. The prevalence of diabetes mellitus has increased, with higher rates among men as compared to women, and this may be an underlying cause of our findings [3]. When comparing the incidence of PAC by race, the overall incidence is the greatest in blacks. Moreover, the incidence in blacks continued to rise at a rapid rate. The risk of pancreatitis is two to three times higher among blacks as compared to whites, and thus our concern is that pancreatitis has a large influence on the incidence differences we found [5]. In whites, the incidence initially increased at a rapid rate as well, but starting in 2012, the rise in incidence plateaued. Among females, overweight adults are considerably higher among black females as compared to whites [3]. Our findings of a continued rise in the incidence of blacks and other races as compared to whites might be an indication that obesity also has a large influence on the incidence of PAC. Moreover, compared to blacks and whites, APIs have a lower overall incidence of PAC. This finding may be related to smoking rates, as Asians typically have low smoking rates [3]. Tobacco smoking is a well-established risk factor for pancreatic cancer, with estimations of a two-fold risk of pancreatic cancer in smokers as compared to non-smokers [2,12-13].

When comparing the incidence of PAC by stage at diagnosis, we found that not only is distant disease the most commonly diagnosed stage overall but that the incidence continues to rise at a rapid rate. Since 2010, the incidence of regional disease has leveled off. The incidence of localized disease continues to rise, however, the overall incidence is considerably lower than that of distant disease. These findings are consistent with a known progression of pancreatic cancer, where the early stages are usually asymptomatic [2,6]. Given the asymptomatic nature of early disease and our findings of worsening incidence of distant disease, the development of screening tools and lab tests are greatly needed to reduce the burden of this disease and ideally diagnose this cancer earlier.

In this study, we also examined the incidence of pancreatic adenocarcinoma based on regional location within the US. We found that the overall incidence was greatest in the Northeast and Midwest. Moreover, starting in 2011, the incidence rate in the Northeast started to level off and after approximately 2013, the incidence was the greatest in the Midwest. The incidence of PAC in the South is also rapidly increasing. Though the reason for these differences in the incidence of PAC is unclear, it may be secondary to variations in obesity. For example, the South, identified as a high obesity region, was the region with the most rapidly increasing rates of PAC after 2008 [14]. Nonetheless, although obesity appears to be a significant risk factor for regional variation in PAC, further research is needed to help identify other risk factors.

There were several strengths and limitations to our study. A strength of our study was the use of the USCS database, which includes data for all 50 states. Other studies have used the SEER database, which has data on a limited percentage of the US population and likely does not represent the true incidence patterns for cancers like the USCS dataset. A limitation of our study was the inability to stratify data by specific risk factors, such as smoking or obesity, and thus we were unable to determine correlations between these risk factors and incidence rates.

## Conclusions

In our study, we evaluated the incidence of pancreatic adenocarcinoma in all 50 states. We found that the overall incidence was greatest in males, blacks, distant disease, and those in the Northeast and Midwest. Between 2001 and 2015, the incidence in blacks and those with distant disease has continued to rise. Moreover, when looking at regional variations in pancreatic adenocarcinoma incidence, starting in 2013, the incidence became the greatest in the Midwest as compared to other regions. The reasons for these trends are still unclear and require further study; however, it appears that obesity and smoking have a substantial role. Our study, to the best of our knowledge, is the first to evaluate the incidence of pancreatic adenocarcinoma in all 50 states. We found important trends and identified at-risk populations for the development of this deadly cancer. Ultimately, we will need to use our findings to develop surveillance guidelines and, hopefully, one day reduce the burden of this disease.
